# Attitude Toward Sexual Aggression Against Women (ASAW) Scale: Development and Structural Validity

**DOI:** 10.1177/10790632251334754

**Published:** 2025-04-13

**Authors:** Chloe I. Pedneault, Chantal A. Hermann, Danielle M. L. Hawthorn, Kevin L. Nunes

**Affiliations:** 1 Carleton University, Ottawa, ON, Canada

**Keywords:** attitude, sexual aggression, measurement, offense-supportive cognitions

## Abstract

We developed a new measure designed to facilitate research on the potential role of men’s attitude toward sexual aggression against women in gender-based sexual violence: the Attitude toward Sexual Aggression against Women (ASAW) scale. We created a large pool of items, in which participants were asked to evaluate how bad it would be if they engaged in various sexually aggressive behaviors in a variety of scenarios. Three independent samples of men recruited from online panels (*N* = 380, 149, and 322) completed these items. Based on their responses, we retained 13 non-redundant items that had the most variance and covered a wide range of sexually aggressive behaviors (e.g., unwanted sexual touching; non-consensual sex), tactics (e.g., threatening to damage her reputation; using physical force), and contexts (e.g., the woman previously agreed to some sexual activity; the woman is intoxicated). An exploratory factor analysis found that all 13 ASAW items loaded strongly onto one factor, which suggests a unidimensional structure. If future research finds evidence for the construct validity of its scores, potential uses for the ASAW include risk assessment, treatment-related attitude-change, and research into the potential causal role of attitudes in sexual aggression against women.

The World Health Organization (2021) estimates that 30% of women aged 15 and older have experienced intimate partner violence (including sexual violence) or sexual violence by a non-partner. In response to this crisis, addressing and preventing sexual violence against women is a priority for many organizations and governments around the world (e.g., Basile et al., 2022; Women and Gender Equality Canada, 2019). *Offense-supportive cognitions* (i.e., cognitions associated with sexually aggressive behavior) have long been thought to play a role in the initiation and maintenance of sexual aggression against women (see Ó Ciardha & Ward, 2013; Szumski et al., 2018 for reviews). Offense-supportive cognitions encompass a wide range of cognitions, such as rape myth acceptance (e.g., Trottier et al., 2021), cognitive distortions (e.g., Ó Ciardha & Ward, 2013), and attitudes toward rape (e.g., Nunes et al., 2018). Evidence suggests that offense-supportive cognitions are generally associated with sexually aggressive behavior (Malamuth, 1981; Malamuth et al., 1996; Nunes et al., 2018; Thompson et al., 2011; Trottier et al., 2021; Wegner et al., 2015; Yapp & Quayle, 2018). Consequently, identifying and measuring offense-supportive cognitions is important for better understanding the role that they may play in sexual offending, which could ultimately help to prevent and address sexual violence against women.

In theory, offense-supportive cognitions can be differentially associated with sexual aggression and may serve different functions for the perpetrator (Maruna & Mann, 2006; Szumski et al., 2018). For instance, some cognitions may play an etiological role, whereas others reinforce behavior after an offense has been committed (Szumski et al., 2018). Theory and evidence suggest that attitudes (i.e., favorable or unfavorable evaluations of a psychological object) play a causal role in determining a wide range of behaviors (Ajzen, 1991, 2001; Ajzen et al., 2018; Glasman & Albarracín, 2006; Kraus, 1995; Sheeran et al., 2016), including violent behavior (Anderson & Bushman, 2002; Nunes et al., 2022). Because of their hypothesized causal role in violent behavior, attitudes are often targeted in interventions aimed at reducing violence (e.g., Bonta & Andrews, 2024).

Consistent with this broader literature, preliminary evidence suggests that men’s attitude toward sexual aggression is associated with, and potentially predictive of, sexually aggressive behavior (Hermann et al., 2018; Hermann & Nunes, 2018; Nunes et al., 2013, 2018; Pedneault et al., 2021, 2022). For instance, Nunes et al. (2018) developed the Evaluation of Rape Scale to assess attitudes toward ‘rape’ using sematic differential scales with bipolar anchors (e.g., *negative* vs. *positive*). In a sample of 660 male undergraduate students, more positive attitudes toward rape were significantly associated with more self-reported past sexually aggressive behavior (*r* = .25) and likelihood of engaging in sexual aggression in the future (*r* = .32). To explore attitudes toward a more nuanced conceptualization of sexual aggression (vs. ‘rape’), Hermann and colleagues (2018) modified the Sexual Experience Survey – Tactics First version (SES-TF; Abbey et al., 2005) to assess attitudes toward behaviorally specific acts of sexual aggression. In a prospective study, Hermann and Nunes (2018) found that more positive attitudes, as measured by the evaluation version of the SES-TF, significantly predicted self-reported sexually aggressive behavior during a four-month follow-up period.

However, despite providing preliminary evidence for the association between attitudes and sexually aggressive behavior, measures of attitude toward sexual aggression developed to date have important limitations. For instance, scores on these measures are extremely positively skewed, such that most men report only very unfavorable evaluations of sexually aggressive behavior (Hermann et al., 2018; Pedneault et al., 2022). We suspect that this largely reflects reality (e.g., Ricciardelli & Moir, 2013)—especially for more extreme sexual aggression (e.g., physical violence vs. psychological coercion). However, such floor effects (i.e., a large proportion of respondents scoring near the lower-boundary of the scale) limit a measure’s ability to detect within-individual and between-individual variance. Thus, if there is true uncaptured variability in attitudes toward sexual aggression, a measure that is designed to capture these differences would be more useful than one that captures less nuance. Indeed, measures that have been developed to assess attitudes toward sexual aggression to date are likely to have gaps in construct coverage as they have not been empirically derived from a large pool of potential items. To ensure good construct coverage, scale development should begin with an overly inclusive pool of items from which the best subset is selected to reflect the construct of interest (Clark & Watson, 2019). Finally, many items have been found to be redundant (e.g., correlations > .90; Pedneault et al., 2021), which unnecessarily increases the length of the measure and complicates certain statistical analyses that are essential for assessing structural validity (e.g., factor analysis; Clark & Watson, 2019; Flake et al., 2017).

The purpose of this article is to introduce a new measure designed to facilitate more rigorous research on men’s attitude toward sexual aggression against women: the Attitude toward Sexual Aggression against Women (ASAW) scale. We decided to focus exclusively on sexual aggression by men against woman because (a) the majority of sexual violence is perpetrated by men against women (e.g., Basile et al., 2022; Cotter & Savage, 2019) and (b) we wanted to maximize the precision and efficiency of our measure and studies. This is not to minimize the problem of sexual violence committed by and against other groups, such as sexual violence by women and against men (e.g., Stemple et al., 2017), as well as sexual violence by and against sexual and/or gender minorities (e.g., McCauley et al., 2018). This approach is rather an effort to focus our finite resources on one form of sexual violence, while acknowledging that more work is required to expand attitude measures to other forms of sexual violence in the future. For instance, work has begun on the development of a new measure of attitudes toward sexual offending against children (Nunes et al., 2023).

Additionally, we chose to develop the ASAW for use with men from the general population rather than focusing on correctional or forensic populations. Research suggests that men from the general community likely represent the largest population of sexually aggressive individuals. That is, because most sexual assaults are not reported to police (e.g., Conroy & Cotter, 2017) and even fewer end with a conviction (e.g., Rotenberg, 2017), few sexually aggressive men actually find themselves in correctional or forensic institutions. When samples of North American men recruited from the community are surveyed, upwards of 40% report engaging in some form of sexual aggression, with approximately 5–10% reporting behavior consistent with sexual assault (e.g., Abbey et al., 2007; Abbey et al., 2011; Hermann et al., 2018). This suggests that interventions aimed at reducing sexually aggressive behavior should include men from the general population.

This article describes the development of the ASAW, including an overview of item development and selection across three independent samples of men recruited from an online pool of research participants. Additionally, the underlying factor structure of the ASAW is explored to identify any items that may not reflect the intended construct and establish whether the measure should have subscales. Empirical tests of the ASAW’s validity are presented separately. The ASAW has the potential of advancing research on the role that attitudes may play in sexual violence against women, as well as clinical uses, including risk assessment and the measurement of attitude change following intervention.

## Method

### Participants

Three independent samples of heterosexual men (18 years or older) living in Canada or the United States were recruited through Qualtrics from an online panel of research participants (Qualtrics, 2020). Specifically, we paid Qualtrics and its partners to recruit participants, who were in turn compensated for completing the study. Each partner has its own approach to compensating panelists, but awards can be gift cards, loyalty points, entry into draws for prizes, and cash awards. As many participants were recruited for each sample based on the resources we had available at the time. Detailed information on the inclusion criteria and demographic characteristics for each sample can be found in Table 1.

**Table 1.** Inclusion Criteria and Sample Characteristics.

Sample	Description of Inclusion Criteria and Sample Characteristics
Sample 1	A total of 957 heterosexual men participated in the first survey. Heterosexual orientation was determined from the demographic question “Who are you most sexually attracted to?” and participants had to endorse “mostly sexually attracted to women” to be included in the study. Of these participants, 128 withdrew from the study and 55 did not otherwise complete the study. Of the 774 remaining men, 0.8% (*n* = 6) were excluded for speeding (i.e., completing the study faster than 1/3 the median time others took to respond to the survey) and 50.1% (*n* = 388) were excluded because they failed more than one of three instructional attention-check questions embedded throughout the survey (e.g., You’re with a woman who is wearing a sexy dress and has been flirting with you. Please select a bit good from the options below to demonstrate that you are paying attention).
	Instructional attention-check questions are designed to detect careless responding and lack of comprehension. Evidence suggests that filtering out those respondents who demonstrate careless responding can improve data quality (e.g., Shamon & Berning, 2020). Although the proportion of participants who failed the attention-check questions in this sample appears quite high (50% of the sample), it is within the range observed in other studies that have used attention-checks (see Thomas & Clifford, 2017 for a review).
	The final sample consisted of 380 participants who were on average 31.2 (SD = 10.2) years old, ranging from 18 to 75. Sixty-five percent (*n* = 247) were from the United States and 35% (*n* = 133) were from Canada. Approximately half were single (52.9%, *n* = 201), 25.8% (*n* = 98) were married, 9.7% (*n* = 37) were in a romantic relationship, 8.9% (*n* = 34) were living with a romantic partner, and 2.6% (*n* = 10) were separated, divorced, or widowed. Participants who were excluded for failing more than one attention-check question did not significantly differ from the final sample in terms of age, *t*(884) = 1.06, *p* = .288, country of residence, *ϕ* = −.06, *p* = .059, or relationship status, *ϕ* = .03, *p* = .967. Race/ethnicity and level of educational attainment was not collected for this sample.
Sample 2	A total of 239 heterosexual men participated in the second survey. Forty-two withdrew and 29 did not complete the study. Of the 168 men who completed the study, 6.6% (*n* = 11) were excluded for speeding and 4.8% (*n* = 8) were excluded because they failed more than one of the three attention-check questions designed to identify careless responding and lack of comprehension.
	The instructional attention-check questions presented to Sample 2 (e.g., Please select “a bit positive”. This is just to check if people are paying attention) were easier/more obvious than those used in Sample 1 (e.g., You’re with a woman who is wearing a sexy dress and has been flirting with you. Please select a bit good from the options below to demonstrate that you are paying attention.), which may explain why a smaller proportion of participants failed the attention-check in Sample 2 (5% vs. 50% in Sample 1). The final sample consisted of 149 participants. For this sample, we set age quotas to recruit mostly younger men: no maximum on participants aged 18–30, a maximum of 25% of participants aged 31–40, a maximum of 15% of participants aged 41–50, and a maximum of 10% of participants aged 51 or older. These quotas were met.
	On average, participants were 30.5 (*SD* = 9.7) years old, ranging from 18 to 75. Additionally, 49% (*n* = 73) were living in the United States and 51% (*n* = 76) were living in Canada. The majority identified as White (70.5%, *n* = 105), 14.1% (*n* = 21) identified as Asian, 5.4% (*n* = 8) Hispanic, 4.0% (*n* = 6) Black, 2.7% (*n* = 4) Arab, 1.3% (*n* = 2) Indigenous, 1.3% (*n* = 2) East Indian, and 0.7% (*n* = 1) Iranian. More than half were single (56.4%, *n* = 84), 27.5% (*n* = 41) were married, 5.4% (*n* = 8) were in a romantic relationship, 10.1% (*n* = 15) were living with a romantic partner, and 0.7% (*n* = 1) were separated, divorced, or widowed. Most participants completed college or university (61.1%, *n* = 91), 36.9% (*n* = 55) completed high school, and 2.0% (*n* = 3) reported that they did not complete high school. Given the small number of participants who were excluded for failing more than one attention-check question (*n* = 8), we did not statistically compare these participants to those who were retained for analysis.
Sample 3	A total of 787 heterosexual men participated in the third survey; 322 withdrew and 17 did not complete the study. Of the 448 men who completed the study, 1.8% (*n* = 8) were excluded for speeding (i.e., completing the survey in less than 1/3 the median survey completion time) and 26.3% (*n* = 118) were excluded for failing more than one of three instructional attention-check questions (e.g., Data quality is important to us. Please select “not at all bad” to show that you have read this question. We appreciate your continued attention). This resulted in a final sample of 322 participants.
	We used the same age quotas that were used in Sample 2 and these quotas were met. This sample was on average 33.7 years old, with ages ranging from 18 to 87. Most participants identified as White (68.0%, *n* = 219), followed by Asian (15.2%, *n* = 49), Black (8.4%, *n* = 27), Hispanic (4.3%, *n* = 14), East Indian (3.4%, *n* = 11), Arab (1.2%, *n* = 4), Indigenous (0.6%, *n* = 2), and Other (2.7%, *n* = 7); 3.4% identified with more than one group. Of those who indicated their relationship status (*n* = 317), 44.5% (*n* = 141) indicated they were single, 35.0% (*n* = 111) were married, 16.1% (*n* = 51) were in a romantic relationship or living with a romantic partner, and 4.4% (*n* = 14) were separated, divorced, or widowed. Additionally, of the 317 participants who indicated their highest level of education, most indicated they completed college or university (70.3%, *n* = 223), 27.1% (*n* = 86) had completed high school, and 2.5% (*n* = 8) had not completed high school. There were no significant differences between this sample and participants who were excluded for failing more than one attention-check question in terms of age, *t*(438) = 1.41, *p* = .159, country of residence, *ϕ* = .08, *p* = .105, race/ethnicity, *ϕ* = .12, *p* = .656, relationship status, *ϕ* = .10, *p* = .359, or education, *ϕ* = .08, *p* = .250.

### Procedure

Overlapping pools of items were presented to the three samples of men. The same procedure was used for each sample. Men who consented to participate were presented with a demographic questionnaire and the pool of items developed for their respective sample (see Results section for more information on the items developed for each sample). Participants could withdraw from the survey at any point. Once a participant withdrew or completed the survey, they were presented images of nature scenes intended to enhance mood and a debriefing form. This procedure was approved by the authors’ university research ethics board (Clearance #108439).

### Overview of Analyses

The authors take responsibility for the integrity of the data, the accuracy of the analyses, and have made every effort to avoid inflating statistically significant results. The statistical analyses were determined a priori and are described next.

#### Item Development and Selection

Item development and selection was an iterative process. Items were developed to assess favorable or unfavorable evaluations of a wide range of sexually aggressive acts and tactics, many of which were based on items from the Sexual Experience Survey (SES) and its modified forms (Abbey et al., 2005; Koss et al., 1987, 2007; Koss & Gidycz, 1985). The SES was used as a primary source for the development of sexually aggressive scenarios because it has undergone extensive validation for measuring sexually aggressive behavior with various samples of men (Koss et al., 2007). Consistent with best practices, we included behaviorally specific language and avoided the use of slang and expressions when describing sexually aggressive behavior (Koss et al., 2007). Additional items were also inspired by the literature on condom use resistance tactics (e.g., Davis et al., 2014) and non-consensual sharing of sexual images (e.g., Marganski & Melander, 2018). Furthermore, sexually aggressive behaviors were paired with potentially mitigating contexts found to increase the level of blame attributed to victims of sexual assault and decrease the perceived culpability of the perpetrator (e.g., van der Bruggen & Grubb, 2014). We hypothesized that including potentially mitigating factors would reduce the perceived wrongfulness of the sexually aggressive behavior for some individuals and, thus, capture more nuance and variability in men’s attitude toward sexual aggression. The pool of potential items was developed by the first two authors and their content reviewed by the last author, all of whom have extensive expertise in the measurement of cognitions related to sexual aggression and attitudes more specifically. Given that the items were heavily based on validated descriptions of sexually aggressive behavior from the SES, the pool of items was not sent for a broader content validity review.

#### Factor Analysis

Next, exploratory factor analysis (EFA) was used to explore the underlying structure of the ASAW. The EFA was conducted in MPlus version 8.4 (Muthén & Muthén, 2017). Factors were extracted from a polychoric correlation matrix using robust weighted least square estimation (i.e., WLSMV estimator). Pearson product-moment correlations tend to underestimate the degree of association between ordinal variables because data variability is reduced relative to continuous data (Flora & Curran, 2004; Holgado-Tello et al., 2010). In contrast, polychoric correlations more accurately estimate correlations between ordinal data and are more robust to violations of normality. Additionally, robust weighted least square estimation is the recommended extraction method for categorical indicators as it does not make distributional assumptions (Kline, 2016; Schmitt, 2011). No attempt was made to impute missing data as the WLSMV estimator computes the model parameters using all available data.

Three factor retention methods were considered when selecting the number of factors to retain: (a) Kaiser criterion, (b) parallel analysis, and (c) minimum average partial (MAP) test. According to the Kaiser criterion, only factors that explain more variance than a single item (i.e., factors with eigenvalue greater than one) should be considered for retention (Kaiser, 1960). Parallel analysis involves generating a series of random datasets with the same sample size and number of variables as the original data (O’Connor, 2000). The number of factors to retain was determined by how many eigenvalues in the original dataset are greater than the 95th percentile of the distribution of randomly generated eigenvalues. Velicer’s (1976) MAP test involves comparing the amount of systematic variance to the amount of unsystematic variance present in the correlation matrix after each factor has been extracted. The number of factors to retain is determined by the number of factor extractions that result in the smallest average squared partial correlation (O’Connor, 2000). Research suggests that parallel analysis and the MAP test are the most accurate methods for determining the number of factors to retain (Schmitt, 2011).

When selecting the final number of factors to retain, we also examined the pattern of standardized factor loadings on each of the extracted factors. Factor loadings ≥ .40 were considered to load onto a factor, with this threshold being the minimum proposed threshold for retaining an item (Matsunaga, 2010). For models with multiple factors, factors were rotated using oblique rotation (Geomin rotation) to improve the interpretability of the factor loadings. Unlike orthogonal rotation, oblique rotation allows factors to correlate, which is important when examining psychological constructs that are likely to be associated. All extracted models were also evaluated using the following three model fit indices: (a) root mean square error of approximation (RMSEA), (b) comparative fit index (CFI), and (c) standardized root mean square residual (SRMR). RMSEA and SRMR are considered badness-of-fit measures, such that larger values indicate poorer fit. RMSEA and SRMR values > .10 may indicate poor fit (Kline, 2016). CFI is considered a goodness-of-fit measure, such that larger values indicate better fit. CFI values greater than .95 are commonly considered to indicate acceptable fit (Hu & Bentler, 1999).

## Results

### Sample 1

Sample 1 was presented with an initial pool of 153 items. The scenarios described in these items were primarily based on the sexual acts and coercive tactics that appear in the SES and its modified forms (Abbey et al., 2005; Koss et al., 1987, 2007; Koss & Gidycz, 1985). For instance, scenarios involved either kissing or sexual touching, oral sex, or sex, with each sexual act being paired with one of the following coercive tactics: telling a woman you’ll make something bad happen to her reputation; intimidating her; physically blocking her from getting away; taking advantage of her when she is drunk or high; threatening to physically harm her; and using physical force. Potentially mitigating factors were also included with each item to introduce more nuance to the sexually aggressive scenarios. Potentially mitigating factors included the woman wearing a sexy dress and flirting, the woman being drunk or high, and the woman being the respondent’s date/girlfriend/wife. Some example items include:

• Your date/girlfriend/wife refuses to let you kiss or sexually touch her, so you tell her that you’ll make something bad happen to her reputation or employment if she doesn’t let you kiss or sexually touch her.
• You’re with a woman who is drunk or high. She refuses to give you oral sex, so you intimidate her by yelling, swearing, or breaking stuff until she gives you oral sex.
• You’re with a woman who is wearing a sexy dress and has been flirting with you. She refuses to have sex with you, so you block her from getting away from you (for example, by blocking the doorway) until she has sex with you.
• You having sex with your date/girlfriend/wife when she is too drunk or high to know or stop what is happening.
• You’re with a woman who is drunk or high. She refuses to give you oral sex, so you physically force her (for example, by holding her down) to give you oral sex.

Together, items consisted of 51 unique scenarios fully crossed with three 4-point bipolar response scales (hence the pool of 153 items). The response scales were selected to capture favorable or unfavorable evaluations of the behavior described in each item: (a) *very bad*-*very good*, (b) *very negative*-*very positive*, and (c) *very sad*-*very fun*. The first two response scales have been shown to load highly on an evaluation component in Osgood et al.’s (1957) foundational work on the measurement of meaning, which divided meaning into three components, namely, evaluation, potency, and activity. The response scale *very sad*-*very fun* was included on an exploratory basis as an alternative to the more commonly used evaluative anchors of *unpleasant-pleasant*, which we believed could be conflated with sexual arousal. Furthermore, we wanted to avoid anchors that could be conflated with societal norms, such as *immoral-moral*, which may capture a different construct from one’s own attitude.

The primary goals driving item selection were two-fold: (a) minimize redundancy across items and (b) reduce floor effects. Item selection began with examining the inter-item polychoric (used for ordinal variables) correlation matrix containing all 153 items. Originally, correlations of ≥ .85 were examined to identify redundant items, but this cut-off resulted in the retention of only five non-redundant items. Therefore, to increase the number of retained items, the cut-off was increased to .90. For each bivariate correlation ≥ .90, only the item with the highest variance was retained. Higher variance was synonymous with a larger proportion of the sample selecting a response other than the least favorable; thus, retaining only items with higher variance was intended to minimize floor effects. This process resulted in a total of 12 items with bivariate polychoric correlations ranging from .67 to .898. All items were positively skewed, with 51% of respondents selecting only the least favorable response option (i.e., *very bad/very sad/very negative*) for all 12 items. With the intention of expanding construct coverage and reducing floor effects, a new pool of items was developed and tested with a second sample of men.

### Sample 2

Sixty-three new scenarios were developed for Sample 2 and fully crossed with two 4-point bipolar response scales (*very bad*-*very good* and *very negative*-*very positive*). This resulted in a total of 126 items. We dropped the *very sad*-*very fun* response scale presented to Sample 1 because it performed similarly to the other response scales and could potentially be conflated with sexual arousal. The new scenarios involve additional sexual acts, such as taking sexual pictures without consent, unwanted sexual touching (leg, butt, and breasts), purposely breaking or removing a condom during sex, and unwanted anal sex. Additionally, two new coercive tactics were tested in this pool of items, namely, threatening to spread rumors and threatening to post sexual pictures on the Internet. Finally, with the goal of reducing floor effects, we tested two additional potentially mitigating factors: the woman engaging in some sexual activity before refusing to engage in other sexual acts and the respondent getting drunk or high with the woman before engaging in coercive tactics. Examples of the new scenarios developed for Sample 2 include:

• You’re with a woman who is wearing a sexy dress and has been flirting with you. She’s letting you kiss and sexually touch her but refuses to give you oral sex, so you push her head down towards your penis until she gives you oral sex.
• You’re drinking or getting high with your date/girlfriend/wife. She’s having sex with you but refuses to let you take sexual pictures of her. When she is too drunk or high to know or stop what is happening, you take sexual pictures of her anyway.
• You’re with a woman who is wearing a sexy dress and has been flirting with you. You put your hand on her leg, but she pushes it away. You put your hand back on her leg.
• You’re with a woman who is drunk or high. She’s refusing to have sex with you, so you tell her that you’ll spread rumours about her if she doesn’t have sex with you.

Item selection prioritized (a) increasing construct coverage, (b) reducing inter-item redundancy, and (c) reducing floor effects. First, items were divided into 12 categories based on the type of coercive tactic they addressed (e.g., taking advantage of a woman when she is drunk or high; using physical force). This modification was made to ensure a more diverse and balanced group of tactics was included in the final measure. Additionally, a stricter threshold for identifying redundancy was used in the remaining samples (i.e., bivariate correlations ≥ .85) as a wider range of correlations was observed between the newly developed items (compared to Sample 1 for which most bivariate correlations were between .85 and .90). Starting with the tactic with the highest variance, the item with the highest variance from each tactic was selected for retention, with the exception that it was not redundant with the previously retained items. To illustrate, the item “You kissing or sexually touching your date/girlfriend/wife when she is too drunk or high to know or stop what is happening” had the highest variance and was retained first, followed by the item “Your date/girlfriend/wife agrees to have sex with you, but only if you wear a condom. You put a condom on. While you’re having sex, you purposely break or remove the condom without her knowing and continue to have sex with her without a condom”. Examples of items that had the lowest variance and that were not retained include “Your date/girlfriend/wife is giving you oral sex but refuses to have sexual intercourse, so you threaten to physically harm her if she doesn’t have sex with you” and “Your date/girlfriend/wife is refusing to have sex with you, so you tell her that you’ll spread rumours about her if she doesn’t have sex with you”.

The process was repeated twice, such that a maximum of two items were retained from each tactic. This resulted in a subset of 13 items reflecting 10 different coercive tactics. Bivariate polychoric correlations between the items ranged from .45 to .849, which suggests less inter-item redundancy relative to Sample 1. However, items remained highly skewed (floor effect), with 48% of participants selecting only the most unfavorable response option (i.e., *very bad/very negative*) for all 13 items.

Further examination of response distributions revealed that variability in responses to the bipolar scales was generally a matter of degree (e.g., *very negative* vs. *a bit negative*) and not of direction (e.g., *very negative* vs. *very positive*). This is not necessarily surprising given that few people would be expected to evaluate sexual aggression *favorably*. In fact, sexual aggression is almost universally condemned, even in correctional populations (e.g., Ricciardelli & Moir, 2013). Additionally, although certain factors may make some sexually aggressive behavior appear less bad/negative (i.e., difference of degree), they likely would not make them appear good/positive (i.e., difference of direction). Consequently, we hypothesized that a unipolar response scale (e.g., *very bad*-*not at all bad*) may reveal greater variability in attitudes toward sexual aggression than the bipolar response scales used so far (e.g., *very bad*-*very good*). We tested this hypothesis with a third independent sample of men.

### Sample 3

Sample 3 was presented with all the items retained from Sample 1 and Sample 2, as well as a few additional items included to ensure construct coverage. Each of the 29 unique scenarios were fully crossed with two bipolar response scales and two unipolar response scales for a total of 116 items. The bipolar response scales were the following:

a) *very bad*, *a bit bad*, *a bit good*, *very good*
b) *very negative*, *a bit negative*, *a bit positive*, *very positive*

The unipolar response scales were the following:

a) *very bad*, *pretty bad*, *not that bad*, *not at all bad*
b) *very negative*, *pretty negative*, *not that negative*, *not at all negative*

To select the best response scale for the final measure, we examined the response distributions across each of the four scales. Visual inspection of the response distributions of individual items across the four different response scales suggested that the *very bad*-*not at all bad* response scale was less positively skewed than the other response scales. Additionally, an omnibus Friedman test indicated that there was a statistically significant difference in median scores across the four response scales, *χ*^2^(3) = 38.03, *p* < .001. Post-hoc analysis using the Wilcoxon signed-rank test with a Bonferroni correction (a = .05/6 = .008) indicated that the *very bad*-*not at all bad* response scale elicited significantly higher endorsement of more favorable attitudes relative to the other response scales. Therefore, to minimize floor effects and increase the ASAW’s ability to capture variance in attitudes toward sexual aggression, we selected the *very bad-not at all bad* response scale for the final measure.

Using the same item selection strategy as in Sample 2, a final subset of 13 items was selected for the ASAW (see Table 2 for a full list of items). Inter-item polychoric correlations ranged from .50 to .848 (see Table 3 for item-level descriptives and correlations). Internal consistency between the items was also high (McDonald’s Omega [ω] = .93). Additionally, floor effects were slightly reduced relative to Sample 1 (51%) and Sample 2 (48%), with a low of 45% of participants endorsing only the most unfavorable response option for all 13 items. The measure with instructions can be found in the Supplemental Appendix.

**Table 2.** Exploratory Factor Analysis of ASAW Items, One-Factor Solution.

	ASAW Item	Factor Loading
1	You’re with a woman who is wearing a sexy dress and has been flirting with you. She’s refusing to let you kiss or sexually touch her, so you block her from getting away from you (for example, by blocking the doorway) until she lets you kiss or sexually touch her.	.88
2	You’re with a woman who is drunk or high. She’s having sex with you but refuses to let you take sexual pictures of her. You take sexual pictures of her anyway.	.86
3	You’re talking with a woman you just met. You put your hand on her leg, but she pushes it away. You put your hand back on her leg.	.79
4	You have oral sex with your date/girlfriend/wife when she is too drunk or high to know or stop what is happening.	.79
5	You’re with a woman who is wearing a sexy dress and has been flirting with you. She’s giving you oral sex but refuses to have sexual intercourse with you, so you tell her that you’ll spread rumours about her if she doesn’t have sex with you.	.86
6	Your date/girlfriend/wife agrees to have sex with you, but only if you wear a condom. You put a condom on. While you’re having sex, you purposely break or remove the condom without her knowing and continue to have sex with her without a condom.	.83
7	You’re with a woman who is wearing a sexy dress and has been flirting with you. She refuses to give you oral sex, so you tell her that you’ll make something bad happen to her reputation or employment if she doesn’t give you oral sex.	.88
8	You’re with a woman you just met who is wearing a sexy dress and has been flirting with you. You grab her butt, but she pushes your hand away. You grab her butt again.	.83
9	Your date/girlfriend/wife refuses to let you kiss or sexually touch her, so you intimidate her by yelling, swearing, or breaking stuff until she lets you kiss or sexually touch her.	.90
10	You have sex with a woman who was flirting with you and wearing a sexy dress when she is too drunk or high to know or stop what is happening.	.82
11	Your girlfriend/wife is refusing to have sex with you, so you tell her that you’ll post sexual pictures of her on the Internet if she doesn’t have sex with you.	.87
12	Your date/girlfriend/wife refuses to give you oral sex, so you physically force her (for example, by holding her down) to give you oral sex.	.87
13	You’re having vaginal sex with your date/girlfriend/wife. You push your penis against her anus to have anal sex with her, but she moves your penis away and says she doesn’t want to have anal sex. You have anal sex with her anyway.	.78

Notes. Sample 3, *N* = 322.

**Table 3.** ASAW Inter-Item Polychoric Correlations and Item-Level Responses.

ASAW Item	1	2	3	4	5	6	7	8	9	10	11	12	13
1	1												
2	.733	1											
3	.651	.729	1										
4	.684	.604	.574	1									
5	.750	.659	.605	.564	1								
6	.747	.729	.589	.718	.736	1							
7	.804	.783	.683	.558	.814	.699	1						
8	.720	.737	.772	.554	.701	.680	.675	1					
9	.787	.727	.712	.668	.825	.729	.813	.708	1				
10	.714	.661	.603	.811	.588	.689	.626	.676	.615	1			
11	.726	.805	.656	.542	.820	.693	.800	.653	.812	.572	1		
12	.848	.722	.616	.612	.728	.719	.707	.699	.798	.661	.748	1	
13	.669	.710	.497	.553	.667	.682	.629	.641	.737	.554	.643	.727	1
*n*	321	322	321	322	322	321	321	321	321	322	322	321	322
*% very bad*	80.4	78.3	66.0	68.0	80.4	77.3	81.9	68.8	81.3	68.3	82.9	84.7	78.0
*% pretty bad*	12.8	17.1	23.4	18.6	14.9	16.8	13.7	20.6	13.7	20.2	12.7	10.9	14.9
*% not that bad*	5.0	3.4	7.5	9.9	3.4	4.4	3.4	8.4	4.0	7.8	3.4	3.4	5.3
*% not at all bad*	1.9	1.2	3.1	3.4	1.2	1.6	0.9	2.2	0.9	3.7	0.9	0.9	1.9

Notes. Sample 3, *N* = 322. Polychoric correlations are used for ordinal variables.

Next, EFA was used to begin examining the underlying structure of the ASAW. The MAP test suggested retaining one factor and the parallel analysis suggested retaining up to three factors. However, the second (eigenvalue = 0.80) and third (eigenvalue = 0.61) factors had eigenvalues less than one, suggesting that they are trivial factors. Nevertheless, we extracted models with one to three factors. The one-factor model fit the data well (RMSEA = 0.08, 90% CI [0.07, 0.09]; CFI = 0.98; SRMR = 0.06), with factor loadings ranging from .78 to .90 (see Table 2). The two-factor (RMSEA = 0.06, 90% CI [0.05, 0.08]; CFI = 0.99; SRMR = 0.04) and three-factor (RMSEA = 0.05, 90% CI [0.03, 0.07]; CFI = 0.996; SRMR = 0.03) models also fit the data well; however, they were less interpretable as they included cross-loadings and Heywood cases (i.e., impossible values). More importantly, they did not meaningfully improve model fit. Therefore, we retained the one-factor model suggesting that the ASAW is unidimensional. This model accounted for 71.6% of the variance in the data for Sample 3.

## Discussion

In this article, we introduced the ASAW as a new measure of men’s attitude toward sexual aggression against women. It asks respondents to evaluate how bad they think engaging in a broad range of sexually aggressive behaviors would be in a variety of scenarios involving themselves and a woman. ASAW items were selected from a large pool of items with the aims of eliminating inter-item redundancy, minimizing floor effects, and improving construct coverage. This resulted in a completely non-redundant set of items covering a wide range of sexually aggressive behaviors and contexts. As a result, the ASAW appears to be more efficient and suitable for statistical analysis than measures previously used to assess attitudes toward sexual aggression (Hermann et al., 2018; Nunes et al., 2013, 2018).

Additionally, floor effects were reduced throughout the various iterations of items tested from Sample 1 to Sample 3, which translates into increased capacity to detect between-person and within-person variance in attitudes toward sexual aggression. Nevertheless, scores remained positively skewed with a substantial proportion of men reporting the lowest possible score (i.e., most unfavorable attitude toward sexual aggression). Of course, if the ASAW is a valid measure of attitudes toward sexual aggression, it is good that a large proportion of men respond in this way. Although this limits the ASAW’s sensitivity for detecting individual differences among a large proportion of men, this begs the question: Do we need to measure differences in attitudes among men who truly hold very unfavorable attitudes toward sexual aggression? We would argue that measuring differences in attitudes beyond this point may be unnecessary because these are not the men who are most likely to engage in sexually aggressive behavior.

With respect to structural validity, results showed that ASAW items all loaded strongly onto one factor, which is consistent with conceptualizations of the attitude construct that imply a unidimensional structure (Ajzen et al., 2018; Fishbein & Ajzen, 1975). For instance, an attitude is commonly defined as a summary evaluation of an attitude object; thus, whereas a person can hold several evaluations relevant to the attitude object, a single attitude is constructed from those evaluations. The current findings are also in line with previous studies that have examined the factor structure of preliminary measures designed to assess attitudes toward sexual aggression (Nunes et al., 2018; Pedneault et al., 2021) and general violence (Nunes et al., 2021).

### Limitations

The samples of men who participated in the current studies were primarily White and highly educated, which may limit the generalizability of the current findings. For example, the distribution of scores on the ASAW may not generalize to correctional populations, which typically have a lower level of education on average. Additionally, it is possible that, had we sampled participants from relatively more violent and/or antisocial populations, scores on the ASAW would have reflected more favorable attitudes toward sexual aggression against women. Nevertheless, research shows that students enrolled in post-secondary education report high levels of sexual victimization and perpetration (e.g., Koss et al., 1987), suggesting that even highly educated samples may demonstrate at least some favorable attitudes toward sexual aggression. Furthermore, we did not test whether the ASAW items function differently across groups, such as races/ethnicities. It is possible that different groups may interpret ASAW items differently, which could create a bias in the assessment of attitudes. These types of questions could be addressed by testing the assumption of measurement invariance between different groups.

As previously mentioned, the ASAW was developed to assess only attitudes toward sexual aggression against women as perpetrated by men, which limits its generalizability with respect to other forms of sexual aggression. For instance, women also engage in sexually aggressive behavior and men are also victims of sexual assault (e.g., Stemple et al., 2017). Furthermore, sexual (e.g., gay, lesbian, pansexual) and gender (e.g., non-binary) minorities represent high-risk groups for sexual victimization (McCauley et al., 2018). Nevertheless, there are differences in beliefs about and perceptions of sexual assault between genders and between different perpetrator-victim gender combinations (e.g., Anderson et al., 1997; Davies et al., 2012; Payne et al., 1999; St George, 2022; Walfield, 2021), which could introduce ambiguity and error variance. In addition, the attitude-behavior relationship is generally stronger when the attitudes are more specific to the behavior in question (e.g., Ajzen et al., 2018; Ajzen & Fishbein, 1977). Extrapolating from this, we would expect that attitudes toward sexual aggression against women would be more strongly associated with sexual aggression against women than would be attitudes toward sexual aggression against other genders. Though these other areas are important, they would be best addressed with separate scales (or subscales). Consequently, the ASAW should only be viewed as a first step in the development of measures of attitude toward sexual aggression. Future research should consider all forms/perpetrators/victims of sexual aggression in the development of additional measures.

Although the ASAW items were developed by researchers with extensive experience measuring and studying offense-supportive cognitions, the items were not sent to other experts for a formal assessment of content validity. It would certainly have been informative to receive input from clinicians, program personnel, and other experts who work in the area of sexual violence prevention or with men who have committed sexual violence. Nevertheless, the wording and structure of the sexually aggressive scenarios were primarily based on well-validated tools, such as the SES and its modified forms. Moreover, the pool of more than 100 unique sexually aggressive scenarios was reduced to only 13 items due to the high intercorrelations between items, indicating a high level of congruence across items. Moreover, all ASAW items ultimately loaded highly onto one factor, which gives us additional confidence in its content validity.

Because the ASAW is a self-report questionnaire, it is possible that participants may be responding in a socially desirable manner, independent of their true attitudes (Paulhus, 1991). There is almost no doubt that social desirability impacts responses on the ASAW to some extent, as sensitive topics are known to be more susceptible to this type of response bias (Krumpal, 2013). However, research also shows that socially desirable responding can be reduced when sensitive surveys are self-administered and when participants are assured of the confidentiality of their data (see Krumpal, 2013 for a review). As each of the current studies consisted of self-administered online surveys and participants were informed in the consent form and throughout the survey that their data would be kept confidential, this may have minimized the impact of social desirability on the results.

### Future Directions and Practical Implications

Future research should explore the extent to which scores on the ASAW reflect a distinct construct from other widely used measures of offense-supportive cognition, and whether they explain unique variance in sexually aggressive behavior. Furthermore, future research should examine the criterion validity of the ASAW, for example, by testing whether scores on the ASAW predict sexually aggressive behaviour, or whether scores on the ASAW reflect more positive attitudes toward sexual aggression among men with a history of sexual offending. Tests of measurement invariance should also be conducted before the ASAW is used for research with different populations, such as men in college or prison.

If future research provides evidence for the construct validity of ASAW scores and these scores are found to predict sexual aggression, then the measure could be used in risk assessment, for example, to identify high-risk cases for secondary or tertiary prevention efforts. Further, if change in ASAW scores is found to predict change in likelihood of sexual aggression, then the ASAW could be used to monitor changes in risk (e.g., Olver & Stockdale, 2020). Finally, if attitudes toward sexual aggression as assessed by the ASAW are found to play a causal role in sexual aggression, then they could be targeted in interventions aimed at preventing sexual aggression, and the ASAW could be used to evaluate progress in those interventions and the effectiveness of those interventions (e.g., Bonta & Andrews, 2024). For these reasons, additional research on the validity of ASAW scores will be crucial in advancing research on attitudes toward sexual aggression.

## Conclusion

In this article, we introduced a new measure designed to assess men’s attitude toward sexual aggression against women. The ASAW is the first measure of its kind to be empirically derived from a large pool of potential items. Preliminary evidence supports the unidimensional structure of the scale, suggesting that responses on the ASAW are driven by a single underlying construct. If future research finds evidence for the construct validity of ASAW scores, potential uses for the scale include risk assessment, treatment-related attitude-change, and research into the potential causal role of attitudes in sexual aggression against women.

## Supplemental Material

Supplemental Material - Attitude toward Sexual Aggression against Women (ASAW) Scale: Development and Structural Validity Supplemental Material for Attitude toward Sexual Aggression against Women (ASAW) Scale: Development and Structural Validity by Chloe I. Pedneault, Chantal A. Hermann, Danielle M. L. Hawthorn, and Kevin L. Nunes in Sexual Abuse
