# Supplementary material for: Attitude Toward Sexual Aggression Against Women (ASAW) Scale: Development and Structural Validity
Source: Sex Abuse. 2025 Apr 13;38(1):23–44. doi: 10.1177/10790632251334754 (PMC12722572; doi:10.1177/10790632251334754)
Supplement: Supplemental Material - Attitude toward Sexual Aggression against Women (ASAW) Scale: Development and Structural Validity [file sj-pdf-1-sax-10.1177_10790632251334754.pdf]

Appendix

Attitude toward Sexual Aggression against Women (ASAW) Scale

The ASAW is designed to measure men's attitude toward their own (real or hypothetical) sexual aggression against women.

RESPONSE SCALES: very bad, pretty bad, not that bad, not at all bad

INSTRUCTIONS: Please rate how you think about having sexual contact with a woman in each of the ways described below.

#	Item
1	You're with a woman who is wearing a sexy dress and has been flirting with you. She's refusing to let you kiss or sexually touch her, so you block her from getting away from you (for example, by blocking the doorway) until she lets you kiss or sexually touch her.
2	You're with a woman who is drunk or high. She's having sex with you but refuses to let you take sexual pictures of her. You take sexual pictures of her anyway.
3	You're talking with a woman you just met. You put your hand on her leg, but she pushes it away. You put your hand back on her leg.
4	You have oral sex with your date/girlfriend/wife when she is too drunk or high to know or stop what is happening.
5	You're with a woman who is wearing a sexy dress and has been flirting with you. She's giving you oral sex but refuses to have sexual intercourse with you, so you tell her that you'll spread rumours about her if she doesn't have sex with you.
6	Your date/girlfriend/wife agrees to have sex with you, but only if you wear a condom. You put a condom on. While you're having sex, you purposely break or remove the condom without her knowing and continue to have sex with her without a condom.
7	You're with a woman who is wearing a sexy dress and has been flirting with you. She refuses to give you oral sex, so you tell her that you'll make something bad happen to her reputation or employment if she doesn't give you oral sex.
8	You're with a woman you just met who is wearing a sexy dress and has been flirting with you. You grab her butt, but she pushes your hand away. You grab her butt again.
9	Your date/girlfriend/wife refuses to let you kiss or sexually touch her, so you intimidate her by yelling, swearing, or breaking stuff until she lets you kiss or sexually touch her.
10	You have sex with a woman who was flirting with you and wearing a sexy dress when she is too drunk or high to know or stop what is happening.
11	Your girlfriend/wife is refusing to have sex with you, so you tell her that you'll post sexual pictures of her on the internet if she doesn't have sex with you.
12	Your date/girlfriend/wife refuses to give you oral sex, so you physically force her (for example, by holding her down) to give you oral sex.
13	You're having vaginal sex with your date/girlfriend/wife. You push your penis against her anus to have anal sex with her, but she moves your penis away and says she doesn't want to have anal sex. You have anal sex with her anyway.

Note. In our studies, we have been randomizing the order in which the items are presented.

SCORING: Responses to each item are scored from 1 to 4 (e.g., *very bad* = 1, *pretty bad* = 2, *not that bad* = 3, *not at all bad* = 4), and averaged to compute the total score.
